# Persistent Monocytic Bioenergetic Impairment and Mitochondrial DNA Damage in PASC Patients with Cardiovascular Complications

**DOI:** 10.3390/ijms26104562

**Published:** 2025-05-09

**Authors:** Dilvin Semo, Zornitsa Shomanova, Jürgen Sindermann, Michael Mohr, Georg Evers, Lukas J. Motloch, Holger Reinecke, Rinesh Godfrey, Rudin Pistulli

**Affiliations:** 1 Vascular Signalling, Molecular Cardiology, Department of Cardiology I, Coronary and Peripheral Vascular Disease, Heart Failure, University Hospital Münster, 48149 Münster, Germany; holger.reinecke@ukmuenster.de; 2 Department of Cardiology I, Coronary and Peripheral Vascular Disease, Heart Failure, University Hospital Münster, 48149 Münster, Germany; rudin.pistulli@ukmuenster.de; 3 Interdisciplinary Heart Failure Section, Department of Cardiology I, Coronary and Peripheral Vascular Disease, Heart Failure, University Hospital Münster, 48149 Münster, Germany; zornitsa.shomanova@ukmuenster.de (Z.S.); juergen.sindermann@ukmuenster.de (J.S.); 4 Department of Medicine A, Hematology, Oncology, and Pulmonary Medicine, University Hospital Münster, 48149 Münster, Germany; michael.mohr@ukmuenster.de (M.M.); georg.evers@ukmuenster.de (G.E.); 5 Department of Internal Medicine II, Paracelsus Medical University, 5020 Salzburg, Austria; l.motloch@salk.at; 6 Department of Internal Medicine II, Salzkammergut Klinikum, OÖG, 4840 Vöcklabruck, Austria; 7 Department of Cardiology, Kepler University Hospital, Medical Faculty, Johannes Kepler University, 4020 Linz, Austria

**Keywords:** CD14^++^ monocytes, long COVID syndrome, monocytic mitochondrial dysfunction, bioenergetic profile, mtDNA damage, reactive oxygen species (ROS), oxidative stress adaptation, heart failure, seahorse analysis

## Abstract

Cardiovascular complications are a hallmark of Post-Acute Sequelae of Severe Acute Respiratory Syndrome Coronavirus 2 (SARS-CoV-2) infection (PASC), yet the mechanisms driving persistent cardiac dysfunction remain poorly understood. Emerging evidence implicates mitochondrial dysfunction in immune cells as a key contributor. This study investigated whether CD14^++^ monocytes from long COVID patients exhibit bioenergetic impairment, mitochondrial DNA (mtDNA) damage, and defective oxidative stress adaptation, which may underlie cardiovascular symptoms in PASC. CD14^++^ monocytes were isolated from 14 long COVID patients with cardiovascular symptoms (e.g., dyspnea, angina) and 10 age-matched controls with similar cardiovascular risk profiles. Mitochondrial function was assessed using a Seahorse Agilent Analyzer under basal conditions and after oxidative stress induction with buthionine sulfoximine (BSO). Mitochondrial membrane potential was measured via Tetramethylrhodamine Ethyl Ester (TMRE) assay, mtDNA integrity via qPCR, and reactive oxygen species (ROS) dynamics via Fluorescence-Activated Cell Sorting (FACS). Parallel experiments exposed healthy monocytes to SARS-CoV-2 spike protein to evaluate direct viral effects. CD14^++^ monocytes from long COVID patients with cardiovascular symptoms (*n* = 14) exhibited profound mitochondrial dysfunction compared to age-matched controls (*n* = 10). Under oxidative stress induced by buthionine sulfoximine (BSO), long COVID monocytes failed to upregulate basal respiration (9.5 vs. 30.4 pmol/min in controls, *p* = 0.0043), showed a 65% reduction in maximal respiration (*p* = 0.4035, ns) and demonstrated a 70% loss of spare respiratory capacity (*p* = 0.4143, ns) with significantly impaired adaptation to BSO challenge (long COVID + BSO: 9.9 vs. control + BSO: 54 pmol/min, *p* = 0.0091). Proton leak, a protective mechanism against ROS overproduction, was blunted in long COVID monocytes (3-fold vs. 13-fold elevation in controls, *p* = 0.0294). Paradoxically, long COVID monocytes showed reduced ROS accumulation after BSO treatment (6% decrease vs. 1.2-fold increase in controls, *p* = 0.0015) and elevated mitochondrial membrane potential (157 vs. 113.7 TMRE fluorescence, *p* = 0.0179), which remained stable under oxidative stress. mtDNA analysis revealed severe depletion (80% reduction, *p* < 0.001) and region-specific damage, with 75% and 70% reductions in amplification efficiency for regions C and D (*p* < 0.05), respectively. In contrast, exposure of healthy monocytes to SARS-CoV-2 spike protein did not recapitulate these defects, with preserved basal respiration, ATP production, and spare respiratory capacity, though coupling efficiency under oxidative stress was reduced (*p* < 0.05). These findings suggest that mitochondrial dysfunction in long COVID syndrome arises from maladaptive host responses rather than direct viral toxicity, characterized by bioenergetic failure, impaired stress adaptation, and mitochondrial genomic instability. This study identifies persistent mitochondrial dysfunction in long COVID monocytes as a critical driver of cardiovascular complications in PASC. Key defects—bioenergetic failure, impaired stress adaptation and mtDNA damage—correlate with clinical symptoms like heart failure and exercise intolerance. The stable elevation of mitochondrial membrane potential and resistance to ROS induction suggest maladaptive remodeling of mitochondrial physiology. These findings position mitochondrial resilience as a therapeutic target, with potential strategies including antioxidants, mtDNA repair agents or metabolic modulators. The dissociation between spike protein exposure and mitochondrial dysfunction highlights the need to explore host-directed mechanisms in PASC pathophysiology. This work advances our understanding of long COVID cardiovascular sequelae and provides a foundation for biomarker development and targeted interventions to mitigate long-term morbidity.

## 1. Introduction

The ongoing global pandemic of Coronavirus Disease 2019 (COVID-19), caused by the severe acute respiratory syndrome (SARS)-CoV-2 virus, has resulted in a wide range of persistent health complications, collectively termed post-acute sequelae of SARS-CoV-2 (PASC) or “long COVID” [1,2]. Of particular concern are cardiovascular complications, including myocardial injury, arrhythmias and heart failure, which persist in a significant proportion of convalescent patients [3]. While the acute phase of the disease is characterized by hyperinflammation and coagulopathy, the mechanisms driving persistent cardiovascular dysfunction in PASC remain poorly understood [4]. However, emerging evidence suggests that mitochondrial dysfunction may play a crucial role in the pathogenesis of PASC, particularly in relation to cardiovascular sequelae [5]. Mitochondria are pivotal to cellular energy production and perform a vital function in immune cell function, regulating processes such as oxidative phosphorylation (OXPHOS), reactive oxygen species (ROS) production, and apoptosis [6]. In the context of viral infections, including SARS-CoV-2, mitochondria are often targets of viral proteins, leading to disrupted bioenergetics and altered immune responses [7].

Monocytes, which are key mediators of innate immunity and vascular homeostasis, rely heavily on mitochondrial function to sustain their diverse roles in inflammation and tissue repair [8]. During acute cases of SARS-CoV-2, monocytes exhibit significant metabolic reprogramming, marked by reduced spare respiratory capacity (SRC) and defective glycolysis, a phenotype reminiscent of immunometabolic paralysis observed in sepsis. These alterations in monocyte metabolism have been linked to the severity of acute COVID-19 and may contribute to the persistent inflammation observed in PASC [9,10].

Recent studies have highlighted the persistence of mitochondrial abnormalities in long COVID. Diaz-Resendiz et al. reported reduced mitochondrial membrane potential (ΔΨm) in leucocytes of patients with long COVID, suggesting chronic bioenergetic insufficiency [11]. The elevation of cardiac and heart failure biomarkers has been well documented in the acute and long COVID patients [12,13,14,15,16,17] The release of mtDNA from damaged mitochondria has emerged as a potential mechanism linking mitochondrial dysfunction to systemic inflammation and cardiovascular pathology [18]. Circulating mtDNA has been shown to act as a damage-associated molecular pattern (DAMP), activating Toll-like receptor 9 (TLR9) signaling and exacerbating endothelial dysfunction and cardiac fibrosis. Notably, elevated levels of circulating mtDNA have been observed in patients with various cardiovascular diseases, including heart failure, suggesting a potential common pathway in the progression of cardiac dysfunction [19,20,21]. The interplay between mitochondrial dysfunction and oxidative stress is of particular interest in the context of PASC-related cardiovascular complications [22]. Oxidative stress has been implicated in the pathogenesis of various cardiovascular diseases. In long COVID patients, persistent oxidative stress may contribute to ongoing mitochondrial damage and cellular dysfunction, perpetuating a cycle of inflammation and tissue injury [23,24].

Despite the progress made in this field, there are still critical gaps in our understanding of how persistent mitochondrial dysfunction in monocytes contributes to cardiovascular pathology in PASC. Specifically, the relationship between monocytic bioenergetic profiles, mtDNA integrity, and functional cardiac impairment has not been directly assessed in the context of long-term PASC. Furthermore, the ability of long COVID monocytes to adapt to oxidative stress challenges, a key aspect of cellular resilience, remains unexplored.

To address these knowledge gaps, we hypothesized that persistent mitochondrial dysfunction in CD14^++^ monocytes underlies cardiovascular symptoms in PASC, driven by mtDNA damage and impaired oxidative stress adaptation. The objective of the present study was to characterize monocytic bioenergetics, mtDNA integrity and ROS dynamics in long COVID patients with heart failure symptoms, using buthionine sulfoximine (BSO)-induced oxidative stress as a functional challenge. The integration of functional assays with mtDNA damage analysis was undertaken to establish a mechanistic framework that would link immune metabolism to cardiac pathology in long COVID. This approach offers insights into the pathophysiology of PASC-related cardiovascular complications and positions monocytic bioenergetic profiling as a potential biomarker for predicting cardiovascular outcomes in long COVID patients. A comprehensive understanding of the role of mitochondrial dysfunction in PASC may pave the way for targeted therapeutic interventions aimed at restoring cellular bioenergetics and mitigating long-term cardiovascular sequelae.

## 2. Results

### 2.1. Baseline Clinical Characteristics of the Cohorts Used in This Study

The baseline characteristics of the entire cohort of 24 patients are shown in Table 1. For the purposes of this study, 14 patients were defined as long COVID patients with cardiovascular symptoms, while 10 were age-matched controls with similar cardiovascular risk factors. A demographic breakdown of the study groups is outlined in Table 1. The long COVID group and the control group exhibited a comparable proportion of male participants, with 57.1% and 50.0% of the respective groups falling into this category (*p* = 0.666). The mean age was 54 ± 16 years in the long COVID group and 53 ± 16 years in the control group (*p* = 0.944). The distribution of cardiovascular risk factors between the groups was found to be similar, with a history of smoking (57.1% vs. 30.0%, *p* = 0.228), dyslipidemia (42.9% vs. 30.0%, *p* = 0.228), hypertension (57.1% vs. 50.0%, *p* = 0.666), and family history of cardiovascular disease (50% vs. 40.0%, *p* = 0.472) being equally prevalent. No patients in either group had been diagnosed with diabetes. The most striking differences were in symptomatology: Dyspnea was more prevalent in long COVID patients compared to controls (92.9% vs. 0%), and angina pectoris was reported by 50% of long COVID patients but none of the control subjects. These findings underscore the pronounced cardiovascular symptom burden in long COVID patients, despite their comparable baseline cardiovascular risk profiles to the control group.

### 2.2. Monocytic Mitochondrial Dysfunction and Impaired Adaptation to Oxidative Stress in Long COVID Monocytes

Since there are reports of mitochondrial dysfunction in various inflammatory and post-viral conditions, we sought to evaluate whether PASC is associated with persistent alterations in monocytic mitochondrial function.

**Figure 1:**
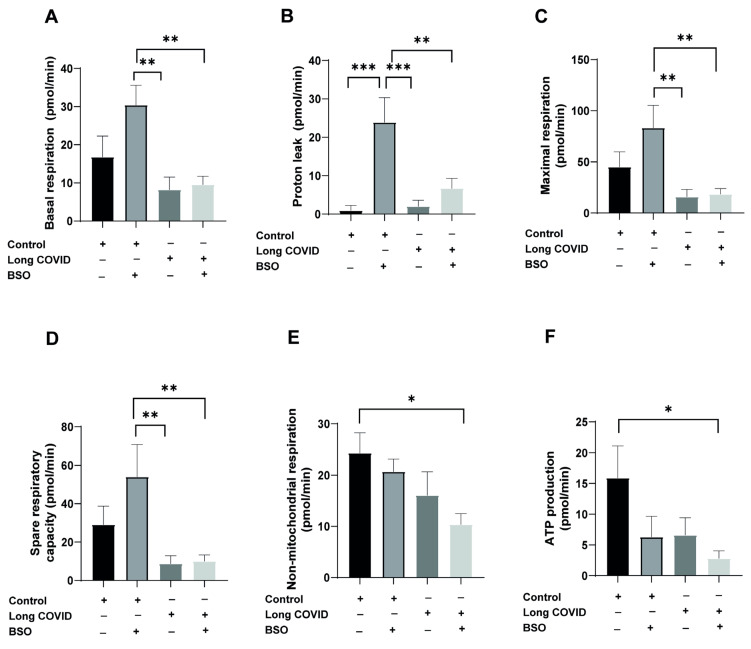
**CD14^++^ monocytes from long COVID patients exhibit impaired mitochondrial function and response to oxidative stress**. CD14^++^ monocytes were isolated from long COVID patients with cardiovascular symptoms (*n* = 14) and age-matched controls with similar cardiovascular risk factors (*n* = 10). Cells were treated with or without BSO (buthionine sulfoximine) for two hours to induce oxidative stress and mitochondrial function parameters were measured using the Seahorse Agilent Analyzer. (**A**) Basal respiration is significantly enhanced in control monocytes after BSO treatment, while long COVID monocytes fail to upregulate basal respiration in response to oxidative stress. (**B**) Proton leak is significantly induced by BSO treatment in control monocytes but not in long COVID samples. (**C**) Maximal respiration is reduced by 65% in long COVID monocytes compared to controls. BSO treatment significantly increases maximal respiration in control monocytes but has no effect on long COVID monocytes. (**D**) Spare respiratory capacity is reduced by 70% in long COVID monocytes. BSO treatment significantly enhances spare capacity in control monocytes but not in long COVID monocytes. (**E**) Non-mitochondrial respiration shows modest differences between groups with significant reduction in long COVID + BSO compared to control. (**F**) ATP production appears reduced in long COVID monocytes compared to controls. All data are presented as means ± SEM. Statistical significance: * *p* < 0.05, ** *p* < 0.01, *** *p* < 0.001.

To assess mitochondrial function, we isolated CD14^++^ monocytes from both PASC and control patients and exposed them to buthionine sulfoximine (BSO) for two hours to induce oxidative stress, followed by comprehensive bioenergetic profiling using the Seahorse Agilent Analyzer (Figure 1). BSO (buthionine sulfoximine) is a specific inhibitor of γ-glutamylcysteine synthetase (γGCS), the rate-limiting enzyme in glutathione (GSH) synthesis [25]. By inhibiting this enzyme, BSO depletes cellular GSH, a major antioxidant that neutralizes reactive oxygen species and maintains cellular redox balance. This depletion leads to increased oxidative stress as cells lose their primary defense mechanism against free radicals, allowing us to examine how monocytes from different groups respond to this oxidative challenge. Our results revealed significant differences in mitochondrial adaptation to oxidative stress between groups.

As shown in Figure 1A, BSO treatment significantly increased basal respiration in control monocytes (from baseline to 30.4 pmol/min), whereas long COVID monocytes failed to upregulate their basal respiration in response to oxidative stress (long COVID + BSO: 9.5 pmol/min vs. control + BSO: 30.4 pmol/min; *p* = 0.0043). Similarly, proton leak, an indicator of mitochondrial membrane integrity, showed a striking 26-fold increase in control monocytes following BSO treatment (from 0.9 to 23.8 pmol/min; *p* = 0.0003), while long COVID monocytes exhibited a non-significant baseline elevation and minimal additional response to BSO challenge (Figure 1B). Maximal respiratory capacity was substantially reduced in long COVID monocytes compared to controls (15.8 vs. 44.9 pmol/min; 65% reduction), though this difference did not reach statistical significance (*p* = 0.4035). Notably, control monocytes responded to BSO treatment with a robust increase in maximal respiration, whereas long COVID monocytes showed no enhancement (Figure 1C). Spare respiratory capacity, representing the bioenergetic reserve critical for adaptation to stress, was 70% lower in long COVID monocytes at baseline (8.7 vs. 29.1 pmol/min; *p* = 0.4143) and showed significant impairment in response to BSO (long COVID + BSO: 9.9 pmol/min vs. control + BSO: 54 pmol/min; *p* = 0.0091) (Figure 1D). While non-mitochondrial respiration showed modest differences between groups (Figure 1E), ATP production appeared reduced in long COVID monocytes compared to controls (6.6 vs. 15.9 pmol/min), although this difference did not reach statistical significance (*p* = 0.2043) (Figure 1F). Collectively, these data indicate that monocytes from long COVID patients exhibit substantial mitochondrial dysfunction, particularly in their ability to adapt their bioenergetic profile in response to oxidative stress, which may contribute to the persistent cardiovascular symptoms observed in these patients.

### 2.3. Impaired Adaptive Response to Oxidative Stress in Long COVID Monocytes

**Figure 2:**
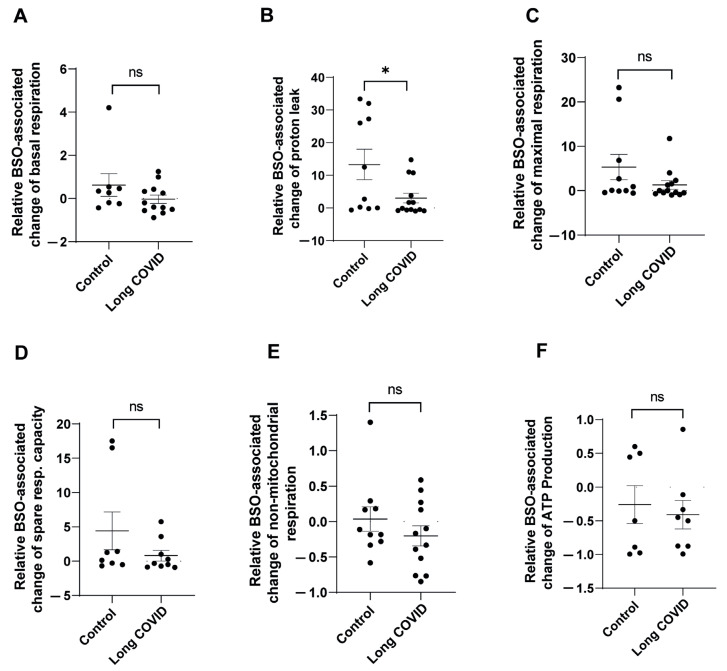
**CD14^++^ monocytes from long COVID patients show impaired relative response to oxidative stress.** CD14^++^ monocytes were isolated from long COVID patients with cardiovascular symptoms (*n* = 14) and age-matched controls with similar cardiovascular risk factors (*n* = 10). Cells were treated with or without BSO (buthionine sulfoximine) for two hours to induce oxidative stress. The relative BSO-associated change in mitochondrial function was analyzed by comparing each parameter between BSO-treated and untreated conditions within each group. (**A**) Relative change in basal respiration shows induction in control monocytes compared to minimal response in long COVID monocytes. (**B**) Relative change in proton leak shows significantly greater induction in control monocytes compared to long COVID monocytes. (**C**) Relative change in maximal respiration shows greater but non-significant induction in control monocytes compared to long COVID monocytes. (**D**) Relative change in spare respiratory capacity reveals enhancement in control monocytes but reduction in long COVID monocytes, though this difference was not statistically significant. (**E**) Relative change in non-mitochondrial respiration and (**F**) relative change in ATP production shows no significant differences between the groups. All data are presented as individual values with means ± SEM. Statistical significance: * *p* < 0.05, ns = not significant.

To further characterize the differences in mitochondrial adaptation to oxidative stress between control and long COVID monocytes, we analyzed the relative BSO-associated changes in mitochondrial function parameters. This approach allowed us to quantify the magnitude of response to oxidative stress in each individual sample, controlling for baseline variations. As shown in Figure 2A, control monocytes demonstrated a robust increase in basal respiration following BSO treatment (mean increase of 62%), whereas long COVID monocytes showed virtually no change in basal respiration (0.3% decrease). Although this differential response appeared substantial, it did not reach statistical significance (*p* = 0.1995).

The most striking difference was observed in proton leak dynamics (Figure 2B). Control monocytes exhibited a dramatic induction of proton leak in response to BSO (13-fold elevation), while long COVID monocytes showed a significantly attenuated response (3-fold elevation), resulting in a statistically significant difference between groups (*p* = 0.0294). Impaired proton leak response in long COVID monocytes likely compromises their ability to mitigate oxidative stress, as proton leak serves as a critical protective mechanism that reduces mitochondrial ROS production when activated. Analysis of maximal respiration (Figure 2C) revealed that control monocytes responded to BSO with a substantial 5.3-fold increase in respiratory capacity, whereas long COVID monocytes showed a blunted response (1.3-fold increase). Despite this apparent difference, the comparison did not reach statistical significance (*p* = 0.1543), likely due to the variability observed within groups. Similarly, spare respiratory capacity (Figure 2D) was markedly enhanced in control monocytes following BSO treatment (4.4-fold increase), while long COVID monocytes not only failed to increase their spare capacity but actually showed a 20% decrease. This differential response, though pronounced, was not statistically significant (*p* = 0.205). The relative BSO-associated changes in non-mitochondrial respiration (Figure 2E) and ATP production (Figure 2F) did not differ significantly between the groups, suggesting that these parameters may be less susceptible to BSO-induced stress or that both groups maintain similar adaptability in these specific aspects of mitochondrial function.

These data demonstrate that long COVID monocytes exhibit impaired adaptive responses to oxidative stress, particularly evident in their inability to appropriately modulate proton leak, a critical mechanism for maintaining mitochondrial membrane integrity during cellular stress.

### 2.4. SARS-CoV-2 Spike Protein Does Not Significantly Alter Mitochondrial Function in Healthy Monocytes

Similar to our findings in long COVID patient monocytes, we hypothesized that direct exposure to SARS-CoV-2 components might impair mitochondrial function in healthy monocytes. Since mitochondrial dysfunction appears to be a hallmark of long COVID syndrome, we investigated whether the viral spike protein alone could recapitulate these effects in vitro. We tested this by exposing CD14^++^ monocytes from healthy donors to recombinant SARS-CoV-2 spike protein S1 for 20 h, with or without subsequent BSO treatment to induce oxidative stress.

**Figure 3:**
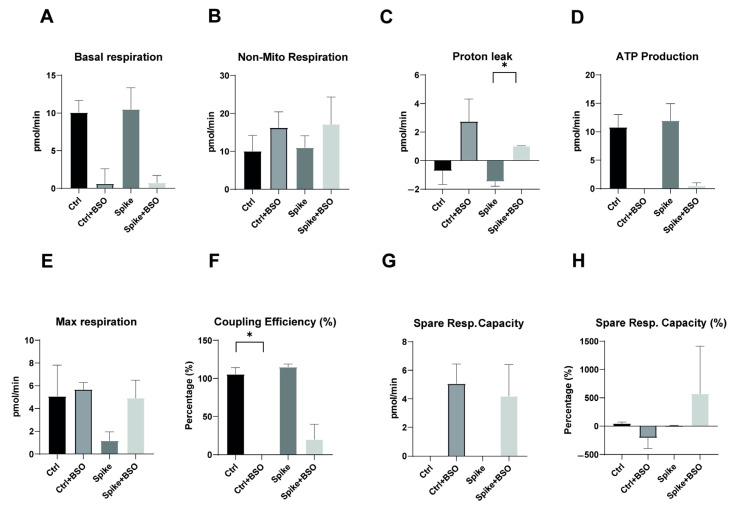
**SARS-CoV-2 spike protein exposure does not substantially alter basal mitochondrial function but affects response to oxidative stress in healthy monocytes.** CD14^++^ monocytes were isolated from three different healthy donors and exposed to recombinant SARS-CoV-2 spike protein S1 for 20 h. Cells were then treated with or without BSO (buthionine sulfoximine) for two hours to induce oxidative stress, and mitochondrial function parameters were measured using the Seahorse Agilent Analyzer with Mito Stress Test Kit. (**A**) Basal respiration shows no significant difference between control and spike-treated monocytes. (**B**) Non-mitochondrial respiration remains relatively consistent across all treatment conditions. (**C**) Proton leak is significantly induced by BSO treatment in control monocytes, but this response is attenuated in spike-treated cells. (**D**) ATP production appears comparable between control and spike-treated monocytes but is markedly reduced with BSO treatment in both conditions. (**E**) Maximal respiration shows modest differences between treatment groups, with spike-treated monocytes demonstrating lower values. (**F**) Coupling efficiency (%) is significantly reduced in spike + BSO treated monocytes compared to control + BSO, suggesting impaired mitochondrial efficiency under oxidative stress. (**G**) Spare respiratory capacity appears to be induced by BSO in both control and spike-treated monocytes. (**H**) Spare respiratory capacity as a percentage shows high variability, particularly in the spike + BSO condition. All data are presented as means ± SEM. Statistical significance: * *p* < 0.05.

As shown in Figure 3A, basal respiration was comparable between control monocytes and spike-protein-treated monocytes, with both groups showing similar reductions following BSO treatment. This suggests that spike protein exposure does not significantly alter baseline oxygen consumption. Similarly, non-mitochondrial respiration remained consistent across all treatment conditions (Figure 3B), indicating that spike protein does not affect oxygen consumption through non-mitochondrial processes. Proton leak, a critical indicator of mitochondrial membrane integrity, showed significant induction in control monocytes following BSO treatment, but this response was notably attenuated in spike-protein-treated cells (Figure 3C, *p* < 0.05). This finding suggests that while spike protein alone does not alter proton leak, it may interfere with the normal proton leak response to oxidative stress. ATP production was comparable between control and spike-protein-treated monocytes (Figure 3D), further supporting that spike protein alone does not directly impair energy production. The maximal respiratory capacity appeared somewhat reduced in spike-protein-treated monocytes compared to controls, though this difference did not reach statistical significance (Figure 3E). However, coupling efficiency, which reflects the proportion of respiratory activity devoted to ATP synthesis, was significantly reduced in spike-protein-treated cells compared to controls (Figure 3F, *p* < 0.05), suggesting subtle impairments in respiratory chain efficiency despite normal ATP production. Spare respiratory capacity following BSO treatment was similar between control and spike-protein-treated monocytes (Figure 3G), although percentage changes in spare capacity showed high variability (Figure 3H). This suggests that while spike protein may not impair the absolute capacity for increased respiration under stress, it might affect the consistency of this response between cells.

Taken together, these data indicate that direct exposure to SARS-CoV-2 spike protein alone does not dramatically alter baseline mitochondrial function in healthy monocytes, but it may subtly modulate specific aspects of respiratory chain activity and stress responses. These modest in vitro effects contrast with the profound mitochondrial dysfunction observed in monocytes from long COVID patients, suggesting that the persistent bioenergetic impairments in long COVID syndrome likely result from complex in vivo processes rather than direct viral protein effects.

### 2.5. Altered Reactive Oxygen Species Response in Long COVID Monocytes

Since we observed impaired mitochondrial function and bioenergetic adaptation in long COVID monocytes, we next investigated whether these cells also display altered reactive oxygen species (ROS) dynamics in response to oxidative stress. ROS production is tightly linked to mitochondrial function and represents a critical indicator of cellular stress responses. To assess this, we isolated CD14^++^ monocytes from long COVID patients and cardiovascular risk-matched controls using negative selection, then exposed these cells to BSO for two hours to induce oxidative stress, and ROS accumulation was measured using FACS.

**Figure 4:**
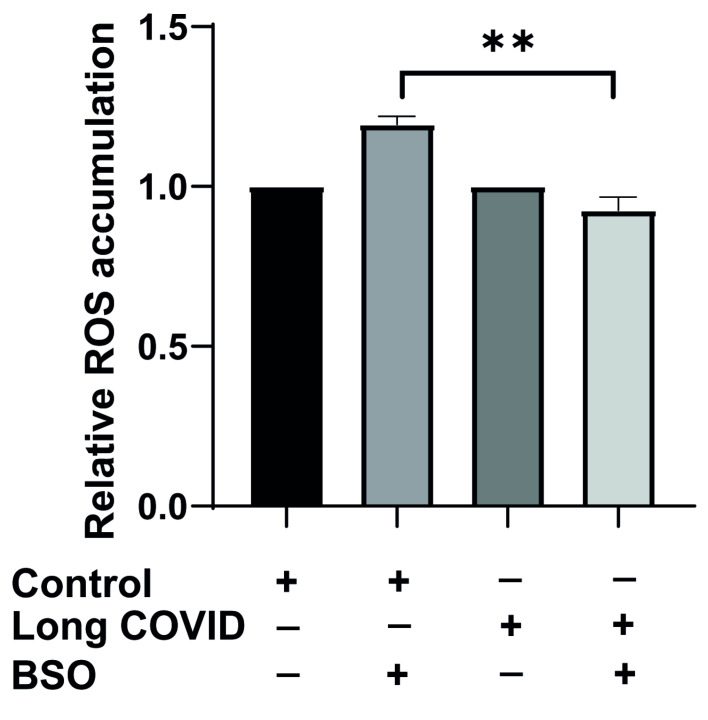
**CD14^++^ monocytes from long COVID patients exhibit impaired reactive oxygen species (ROS) response to oxidative stress.** CD14^++^ monocytes were isolated from long COVID patients with cardiovascular symptoms (*n* = 6) and age-matched controls with similar cardiovascular risk factors (*n* = 4) using a negative selection method. Cells were allowed to rest for two hours or exposed to BSO (buthionine sulfoximine) for two hours to induce oxidative stress, and ROS accumulation was measured using the FACS approach. Basal ROS levels do not differ between control and long COVID monocytes. Control monocytes respond to BSO treatment with increased ROS production, while long COVID monocytes fail to upregulate ROS in response to oxidative stress and instead show a slight reduction. All data are presented as means ± SEM. Statistical significance: ** *p* < 0.01.

As shown in Figure 4, basal ROS levels did not differ significantly between control and long COVID monocytes under resting conditions, suggesting that the baseline oxidative state is comparable between groups. However, when challenged with BSO, a striking difference in ROS dynamics emerged. Control monocytes responded to BSO with the expected increase in ROS production (1.2-fold elevation), indicating a normal oxidative stress response. In contrast, long COVID monocytes not only failed to increase ROS production but actually showed a slight reduction in ROS levels following BSO treatment (6% decrease). This differential response was highly significant (*p* = 0.0015).

### 2.6. Elevated Mitochondrial Membrane Potential in Long COVID Monocytes

**Figure 5:**
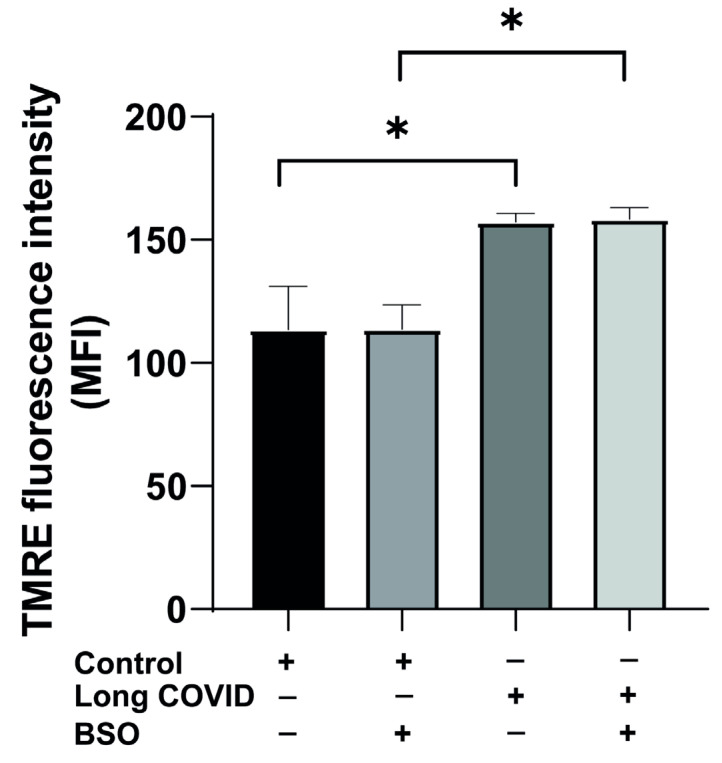
**CD14^++^ monocytes from long COVID patients exhibit elevated mitochondrial membrane potential that remains stable under oxidative stress.** CD14^++^ monocytes were isolated from long COVID patients with cardiovascular symptoms and age-matched controls with similar cardiovascular risk factors. Cells were treated with or without BSO (buthionine sulfoximine) for two hours to induce oxidative stress, and mitochondrial membrane potential was measured using the TMRE (Tetramethylrhodamin, Ethylester, Perchlorat) assay. Long COVID monocytic mitochondria display a significantly elevated mitochondrial membrane potential compared to controls. The induction of oxidative stress by the addition of BSO does not influence the membrane potential in either group. All data are presented as means ± SEM. Statistical significance: * *p* < 0.05.

Mitochondrial membrane potential is a critical indicator of mitochondrial health and function in immune cells. As shown in Figure 5, CD14^++^ monocytes from long COVID patients display altered mitochondrial characteristics compared to control subjects. Monocytes isolated from long COVID patients exhibit significantly elevated mitochondrial membrane potential compared to those from control subjects (157 vs. 113.7 mean TMRE fluorescence, *p* = 0.0179), suggesting a fundamental alteration in mitochondrial bioenergetics that persists after the acute infection. This elevation in membrane potential may reflect a compensatory response to underlying mitochondrial dysfunction or inefficient electron transport chain activity in long COVID monocytes.

To determine whether this altered membrane potential phenotype could be modified by oxidative stress, we treated monocytes with BSO, a glutathione synthesis inhibitor that induces cellular oxidative stress. Interestingly, BSO treatment had a minimal effect on mitochondrial membrane potential in both groups. Control monocytes maintained their baseline membrane potential (113.5 after BSO vs. 113.7 at baseline), while long COVID monocytes similarly maintained their elevated membrane potential (158 after BSO vs. 157 at baseline). The significant difference between groups persisted after BSO treatment (*p* = 0.0179), indicating that the altered mitochondrial membrane potential in long COVID monocytes represents a stable phenotype that is not readily normalized by acute oxidative stress.

This resistance to BSO-induced changes in membrane potential contrasts sharply with our previous findings regarding respiratory parameters and ROS production, where long COVID monocytes showed impaired responses to oxidative stress. The elevated but stable membrane potential in long COVID monocytes, regardless of oxidative challenge, suggests persistent remodeling of mitochondrial function that may contribute to the long-term immune dysregulation and cardiovascular symptoms observed in long COVID syndrome.

### 2.7. Mitochondrial DNA Depletion and Region-Specific Damage in Long COVID Monocytes

**Figure 6:**
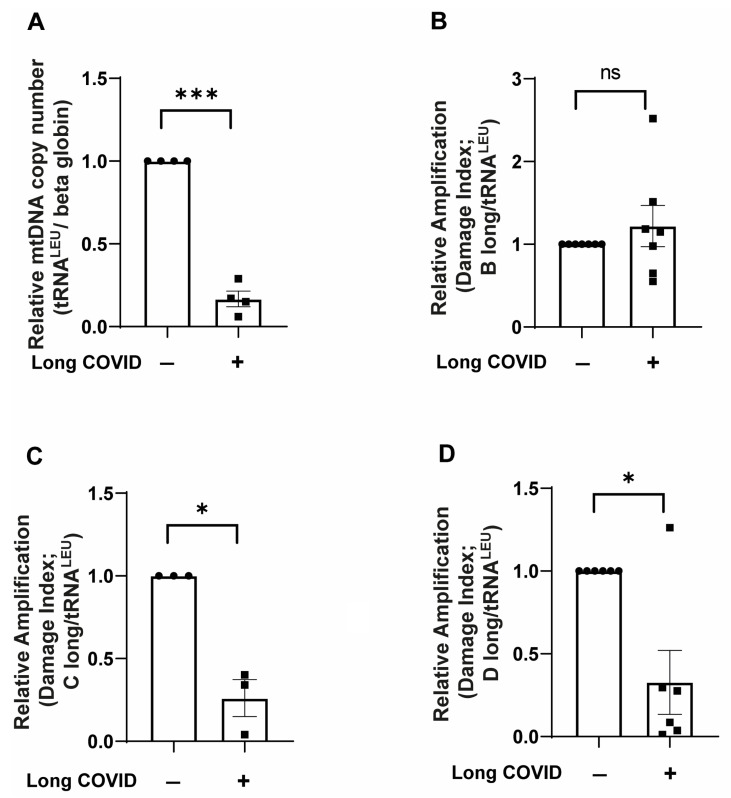
**CD14^++^ monocytes from long COVID patients show significant mtDNA depletion and region-specific damage.** CD14^++^ monocytes were isolated from long COVID patients with cardiovascular symptoms and age-matched controls with similar cardiovascular risk factors. Total DNA was extracted and analyzed for mitochondrial DNA content and integrity using qPCR. (**A**) Relative mtDNA copy number shows significant depletion in long COVID monocytes compared to controls, assessed by the ratio of mitochondrial tRNA^LEU^ to nuclear beta-globin. (**B**) Relative amplification of the B long mtDNA region normalized to the short reference tRNA^LEU^ fragment shows no significant difference between long COVID and control monocytes. (**C**) Relative amplification of the C long mtDNA region normalized to tRNA^LEU^ reveals significant damage in long COVID monocytes compared to controls. (**D**) Relative amplification of the D long mtDNA region normalized to tRNA^LEU^ demonstrates significant damage in long COVID monocytes compared to controls. Lower amplification index values indicate greater mtDNA damage in the respective regions. All data are presented as means ± SEM. Statistical significance: * *p* < 0.05, *** *p* < 0.001, ns = not significant.

Similar to dysfunction observed in various cellular systems under pathological conditions, long COVID syndrome may alter mitochondrial DNA integrity in immune cells, potentially contributing to their impaired function. Since mitochondrial dysfunction is frequently associated with alterations in mitochondrial DNA (mtDNA), we investigated whether long COVID patients exhibit changes in monocytic mtDNA content and integrity. We isolated CD14^++^ monocytes from long COVID patients and age-matched controls, extracted total DNA, and performed detailed analysis of the mtDNA copy number and regional damage. As shown in Figure 6A, long COVID monocytes displayed a dramatic reduction in mtDNA copy number compared to controls. Quantification of the mitochondrial-specific gene tRNA^LEU^ relative to nuclear beta-globin revealed that long COVID monocytes contain approximately 80% less mtDNA compared to control cells (*p* < 0.001). This substantial depletion of mitochondrial genomes likely contributes to the bioenergetic deficits we previously observed in these cells.

We next investigated whether the remaining mtDNA in long COVID monocytes exhibited signs of damage by analyzing the amplification efficiency of three different long mtDNA fragments relative to the short reference fragment tRNA^LEU^. Interestingly, the analysis of region B (Figure 6B) showed no significant difference in amplification efficiency between long COVID and control samples, suggesting that this specific mitochondrial genome region remains relatively intact. However, analysis of region C (Figure 6C) revealed significant damage in long COVID monocytes, with an approximately 75% reduction in amplification efficiency compared to controls (*p* < 0.05). Similarly, region D (Figure 6D) showed significant damage in long COVID samples, with an approximately 70% reduction in amplification compared to controls (*p* < 0.05).

The observed pattern of region-specific mtDNA damage, combined with overall mtDNA depletion, provides molecular evidence for persistent mitochondrial compromise in long COVID monocytes. The regional selectivity of mtDNA damage suggests that certain mitochondrial genome segments may be particularly vulnerable to SARS-CoV-2-associated oxidative stress, potentially affecting the expression of specific mitochondrial genes involved in electron transport chain function and cellular bioenergetics.

## 3. Discussion

The COVID-19 pandemic has unveiled a spectrum of long-term health complications, with cardiovascular manifestations emerging as a critical concern in post-acute sequelae of SARS-CoV-2 infection (PASC) [1,26]. This study provides compelling evidence that persistent monocytic mitochondrial dysfunction and mtDNA damage underpin cardiovascular complications in long COVID patients. Our findings reveal that CD14^++^ monocytes from these patients exhibit profound bioenergetic impairment, defective oxidative stress adaptation, elevated mitochondrial membrane potential, and region-specific mtDNA damage, offering mechanistic insights into the pathophysiology of PASC-related cardiovascular sequelae.

Mitochondria are central to immune cell function, orchestrating energy production, redox balance, and apoptosis [6]. In long COVID monocytes, we observed a striking inability to upregulate basal respiration, maximal respiration, and spare respiratory capacity in response to oxidative stress (Figure 1A–D). These defects mirror the “immunometabolic paralysis” reported in sepsis [10] and align with prior observations of mitochondrial dysfunction in long COVID [9,27]. The failure to induce proton leak—a protective mechanism that dissipates membrane potential to reduce ROS overproduction—suggests a breakdown in mitochondrial stress adaptation (Figure 1B) [28]. This is particularly consequential, as proton leak regulates ROS-mediated signaling and prevents electron transport chain (ETC) overload [29]. The attenuated proton leak response in long COVID monocytes likely exacerbates oxidative damage, creating a vicious cycle of mitochondrial and cellular injury.

The elevated mitochondrial membrane potential (ΔΨm) in long COVID monocytes (Figure 5) further underscores this dysregulation. While ΔΨm typically reflects ETC efficiency, its persistence despite impaired respiration suggests decoupling between proton gradient maintenance and ATP synthesis—a hallmark of mitochondrial uncoupling [29]. Such uncoupling may represent a failed compensatory mechanism to mitigate ROS production, ultimately contributing to bioenergetic failure. This phenomenon has been observed in aging and neurodegenerative disorders but is novel in the context of post-viral syndromes [30]. The elevated mitochondrial membrane potential observed in long COVID monocytes (157 vs. 113.7 TMRE fluorescence units in controls, *p* = 0.0179) represents a significant deviation from normal physiological parameters. Typically, the mitochondrial membrane potential ranges from −150 to −180 mV in healthy cells, with regulated fluctuations between approximately −108 mV during high energy demand and −158 mV during resting states [31]. Our TMRE fluorescence values cannot be directly converted to millivolts without a calibration curve specific to our experimental system, but the relative increase of approximately 38% in long COVID monocytes suggests a hyperpolarized state. Similar hyperpolarization has been observed in heart failure, where altered mitochondrial membrane potential contributes to increased ROS production and cellular dysfunction. This sustained elevation in membrane potential, despite impaired respiratory chain activity, may represent a maladaptive response that contributes to cellular stress and compromised bioenergetics observed in long COVID.

Our discovery of significant mtDNA depletion (80% reduction) and region-specific damage in long COVID monocytes (Figure 6) provides a molecular basis for their bioenergetic deficits. mtDNA is highly vulnerable to oxidative damage due to its proximity to ROS-generating ETC complexes and lack of protective histones [32]. The preferential damage to regions C and D of the mitochondrial genome—which encode critical ETC components [33]—likely disrupts oxidative phosphorylation, exacerbating the respiratory defects observed in Seahorse assays. Circulating mtDNA fragments, acting as DAMPs, can activate TLR9 and fuel systemic inflammation [20], a pathway implicated in myocardial injury and endothelial dysfunction [19]. Elevated mtDNA levels in heart failure patients correlate with disease severity [34], suggesting shared mechanisms between PASC and traditional cardiovascular pathologies. The 80% reduction in mtDNA copy number observed in our long COVID patients is comparable to changes reported in other cardiovascular conditions. Recent studies have demonstrated that a decreased mtDNA copy number is associated with increased risk of cardiovascular events including heart failure [35]. Similarly, in mouse models of heart failure, significant decreases in mtDNA copy number have been observed in cardiomyocytes, with the restoration of mtDNA levels through the overexpression of mitochondrial transcription factor A (mtTFA) resulting in improved cardiac function and survival [36]. In atherosclerosis, reduced mtDNA has been linked to plaque instability and increased risk of adverse cardiac events [37,38]. The magnitude of mtDNA depletion in our long COVID patients suggests a severe level of mitochondrial compromise that may contribute to persistent cardiovascular symptoms and potentially increased long-term cardiac risk.

While we observed significant mitochondrial dysfunction in long COVID monocytes, these cells remained viable during our experimental timeframe, suggesting they can persist despite their bioenergetic impairments. This aligns with evidence that monocytes in severe COVID-19 can undergo pyroptosis, a pro-inflammatory form of programmed cell death involving inflammasome activation and IL-1β release [39]. The persistence of dysfunctional but viable monocytes may contribute to ongoing inflammation and tissue damage in long COVID. Future studies should specifically assess markers of pyroptosis such as caspase-1 activation and GSDMD cleavage in long COVID monocytes to better understand the relationship between mitochondrial damage and cell death pathways in this condition.

The lack of mitochondrial dysfunction in healthy monocytes exposed to SARS-CoV-2 spike protein (Figure 3) contrasts sharply with the profound defects in long COVID patient cells. This contradiction implies that mitochondrial impairment in PASC arises not from direct viral protein toxicity but from maladaptive host responses to long infection. Several potential mechanisms could be responsible for this. First is persistent inflammation where chronic interferon signaling and cytokine release (TNF-α, IL-6) can inhibit mitochondrial biogenesis and promote ROS overproduction [40]. Second could be autoimmunity where molecular mimicry between viral proteins and mitochondrial antigens may drive antibody-mediated mitochondrial damage [41].

The third and most viable mechanism could be connected to metabolic reprogramming where prolonged glycolytic shift during acute infection may “lock” monocytes into a low-energy state, impairing their ability to reactivate oxidative metabolism. While classical monocytes express minimal ACE2, SARS-CoV-2 spike protein can interact with immune cells through alternative receptors including TLR4 [42], CD147 [43], and cell surface GRP78 [44]. Our finding that spike protein exposure alone did not recapitulate the profound mitochondrial dysfunction observed in long COVID patient monocytes provides important mechanistic insight, suggesting that persistent bioenergetic defects arise primarily from maladaptive host responses rather than direct viral protein effects. This directs future therapeutic development toward host-directed approaches for restoring mitochondrial function rather than virus-neutralizing strategies

Our findings on monocyte dysfunction should be considered in the broader context of innate immune dysregulation in COVID-19. Recent research has demonstrated important cross-talk between NK cells and monocytes in COVID-19 pathogenesis [45]. NK cells in severe COVID-19 exhibit a unique profile characterized by both activation and dysfunction, with altered cytokine production and cytotoxicity. This NK cell dysregulation has been shown to be partially mediated by interactions with monocytes, particularly through both soluble factors and direct cell–cell contact. The mitochondrial dysfunction we observed in long COVID monocytes may influence this cross-talk, potentially contributing to persistent immune dysregulation. Future studies should investigate whether the bioenergetic impairments in monocytes affect their ability to properly interact with and regulate NK cell function in long COVID, as this could represent an additional mechanism through which monocyte dysfunction contributes to persistent inflammation and tissue damage.

The bioenergetic failure in long COVID monocytes aligns with the clinical presentation of fatigue, exercise intolerance, and heart failure symptoms in PASC. Mitochondrial dysfunction reduces cellular resilience, impairing tissue repair and amplifying endothelial injury [46].

Targeting mitochondrial health may thus offer therapeutic benefits. Antioxidants like Coenzyme Q10 and MitoQ could mitigate ROS overproduction [47]. Similarly, SGLT2 inhibitors like Empagliflozin, known to improve mitochondrial efficiency in diabetes [48], may restore redox balance in PASC. Enhancing base excision repair pathways or administering mitochondrial-targeted peptides, like SS-31, could address mtDNA damage [49]

While this study provides novel insights, limitations exist. The small cohort size (*n* = 24) and cross-sectional design preclude causal inferences. Future studies should correlate mitochondrial parameters with cardiac imaging (MRI) and biomarkers (NT-proBNP). Another important aspect that is open for investigation areepigenetic modifications (mtDNA methylation) as drivers of persistent mitochondrial dysfunction in long COVID syndrome.

In conclusion, the present study establishes mitochondrial dysfunction as a cornerstone of cardiovascular complications in long COVID syndrome. These complications are characterized by persistent bioenergetic failure, impaired oxidative stress adaptation, and significant mtDNA damage in CD14^++^ monocytes. The finding that these immune cells are unable to modulate proton leak, maintain respiratory flexibility, or preserve mitochondrial genome integrity underscores a profound metabolic paralysis that likely perpetuates systemic inflammation. The findings contribute to our understanding of the pathophysiology of PASC by demonstrating the link between mitochondrial compromise and clinical cardiovascular symptoms, thus highlighting mitochondria as a critical therapeutic target. Consequently, interventions designed to restore redox balance, enhance mitochondrial resilience, or repair mtDNA damage may offer promising strategies to mitigate the long-term consequences of SARS-CoV-2 infection.

## 4. Materials and Methods

### 4.1. Patient Recruitment

Patients recruited in this study had a proven COVID-19 infection three months to one year before admission. COVID-19 disease was previously diagnosed using real-time qPCR. Patients were admitted to our department due to persisting cardiac symptoms, such as angina or dyspnea, in their daily activities. Patients with a medical history of cancer or autoimmune diseases, persistent acute kidney injury, or infections during admission were excluded. Pulmonary diseases were ruled out by clinical examination, pulmonary function test, and, where required, pulmonary imaging. The diagnostic workup further included obtaining patient history and clinical examinations. Through a cardiac work-up covering electrocardiography, echocardiography, functional stress testing, and, when necessary, coronary angiography, structural heart disease was excluded. All recruited patients and matched unaffected controls provided informed consent during their admission to our hospital. The patients were matched with unaffected controls for age, sex, and cardiovascular risk factors (history of smoking, diabetes mellitus, dyslipidemia, hypertension, and family history of cardiovascular diseases).

### 4.2. Monocyte Isolation from Clinical Cohorts and Healthy Individuals

Our study focused on circulating CD14^++^CD16^−^ monocytes as these cells play a critical role in cardiovascular homeostasis and can serve as an accessible biomarker of systemic immune and metabolic dysfunction in long COVID patients with cardiovascular manifestations. CD14^++^CD16^−^ human monocytes were isolated from healthy donors and from control and long COVID patients according to a published protocol [50] using magnet-assisted cell sorting (MACS) with negative selection using Monocyte Isolation Kit II human from Miltenyi Biotec. The study was approved by the scientific and ethics committee of the University of Münster and conforms to the principles of the Declaration of Helsinki. Written informed consent was obtained from all donors by the blood bank, and thrombocyte reduction filters were provided anonymously without sharing personal and detailed information. The purity of isolated cells was confirmed by FACS, using CD14/CD16 staining, and they were around 98% pure.

### 4.3. Monocyte Culture

Primary human monocytes were maintained in RPMI-1640 medium (+L-Glutamine, −D-Glucose, Thermo Scientific, Waltham, MA, USA) supplemented with 5 mM glucose, 10% fetal bovine serum (FBS), and 1% Penicillin/Streptomycin. For migration experiments and signaling studies, cells were starved for 2–4 h in FBS-free medium. Monocytes were kept in an incubator at 37 °C and 5% CO_2_. Normoglycemic medium: RPMI-1640 medium (Gibco, Thermo Scientific, Waltham, MA, USA), Penicillin/Streptomycin, 5 mM glucose, and 20% fetal bovine serum.

### 4.4. Seahorse Analysis of Mitochondrial Function

The Cell Mito Stress Test kit was performed using a Seahorse analyzer according to the manufacturer’s protocol. A total of 300,00 monocytes were seeded per well into a poly-L-lysed-coated XF cell culture mini plate and incubated for 3 h at 37 °C and 5% CO_2_. The cells were cultured in XF-medium (XF Base Medium Minimal RPMI; Agilent Technologies, Santa Clara, CA, USA) containing 10 mM glucose, 2 mM l-glutamine, and 1 mM sodium pyruvate (all from Sigma-Aldrich, St. Louis, MO, USA). After three measurements of baseline recording, oligomycin A, FCCP, and rotenone/antimycin A were injected, followed by three measurement intervals after each injection. For BSO pretreatment, the test compound was added for 2–2.5 h before the assay. The data were analyzed using Agilent Seahorse Analytics (Agilent). The results were plotted using GraphPad Prism software, version 8.

### 4.5. ROS Detection Assay

To assess the cellular redox status, a previously used protocol was used [51]. Roughly, 100,000 cells per donor were used. For redox modulation, cells were treated with BSO for 2 h. CellROX Reagent (Invitrogen, Thermo Fisher Scientific, Waltham, MA, USA) was then added to a final concentration of 5 μM. The cells were incubated for 30 min at 37 °C in the dark. After incubation, the medium was removed, and the cells were washed three times with PBS. Fluorescence intensity was measured using a fluorescence plate reader with an excitation/emission of 485/520 nm (Victor, PerkinElmer, Shelton, CT, USA) and flow cytometry (FITC; excitation/emission: 485/520 nm, Guava easyCyte, Millipore, Darmstadt, Germany).

### 4.6. Mitochondrial Membrane Potential

A total of 125,000 monocytes per donor were used for the assay. The FCCP (carbonyl cyanide-p-trifluoromethoxy phenylhydrazone), an uncoupler of mitochondrial oxidative phosphorylation, was applied at a concentration of 20 μM for 10 min. The cells were incubated with 1 μM TMRE for 20 min at 37 °C in the dark, followed by washing once with 100 μL of PBS containing 0.2.% bovine serum albumin. A volume of 200 µL of PBS containing 0.2% bovine serum albumin was added to each well, and the fluorescence was measured with excitation/emission: 549/575 nm (Victor, PerkinElmer, Shelton, CT, USA).

### 4.7. DNA Damage Analysis and Mitochondrial Copy Number

The total DNA was isolated from 3–5 million monocytes using a Quick DNA mini prep kit (Zymo Research, Irvine, CA, USA), and, after enrichment of the DNA, the measurement of mitochondrial and nuclear DNA damage was performed by RT-qPCR as described previously [52]. In each of the genomic or mitochondrial gene loci examined, a large DNA fragment (3000 to 4000 base pairs) was employed as a sensor for DNA damage, and an internally nested small fragment (50 to 70 base pairs), which is assumed to be undamaged, as a reference. The nested intact reference fragment is effectively amplified by PCR, but the amplification of the large sensor is obstructed by other DNA lesions such as abasic sites, thymine dimers, strand breaks, and oxidative damage. Following the induction of DNA damage, the exponential amplification phase for the damage-sensitive large fragment is achieved at a later time point relative to the nested intact reference. The reactions were carried out using a CFX Connect Real-Time PCR Detection System (Bio-Rad, Feldkirchen, Germany). Samples were measured in duplicates. Cq values were calculated using CFX manager software 3.1, and replicates were averaged. For mitochondrial copy number analysis, the levels of mitochondria-specific gene tRNA^LEU(UUR)^ were measured compared to nuclear DNA specific gene beta globin using RT-qPCR. All primers used are given in Supplementary Table S1.

### 4.8. Statistical Analysis

To analyze the significance of differences in experiments with monocytes isolated from long COVID or healthy individuals, the Mann–Whitney Rank Sum Test (for intergroup comparisons) or Kruskal–Wallis One-Way Analysis of Variance on Ranks with Tukey or Dunn’s post hoc correction was used. For all the other experiments, two-sample independent *t*-tests or, when multiple comparisons were made, Kruskal–Wallis One-Way Analysis of Variance on Ranks with Tukey or Dunn’s post hoc correction was performed. The level of significance was defined as *p* < 0.05. All statistics and graphs were generated using GraphPad Prism 8 software.

## 5. Conclusions

This study establishes mitochondrial dysfunction as a hallmark of PASC-related cardiovascular complications. By linking bioenergetic failure, mtDNA damage, and oxidative stress adaptation defects to clinical symptoms, we propose a paradigm where mitochondrial impairment perpetuates inflammation and tissue injury. Therapeutic strategies targeting mitochondrial resilience may alleviate the long-term burden of long COVID syndrome.

## Figures and Tables

**Table 1:** Baseline clinical characteristics of the cohorts used in this study.

Characteristics	Long COVID (*n* = 14)	Control (*n* = 10)	Overall	*p*-Value
Age—yr (±SD)	54 (±16)	53 (±16)	54 (±16)	0.944
Male sex—no. (%)	8 (57.1)	5 (50.0)	13 (54.2)	0.666
History of smoking—no. (%)	8 (57.1)	3 (30.0)	11 (45.6)	0.228
Diabetes—no. (%)	0 (0)	0 (0)	0 (0)	+
Dyslipidemia—no. (%)	6 (42.9)	3 (30.0)	9 (37.5)	0.228
Hypertension	8 (57.1)	5 (50.0)	13 (54.2)	0.666
Family history of CVD—no. (%)	7 (50)	4 (40.0)	11 (45.6)	0.472
Dyspnea—no. (%)	13 (92.9)	0 (0.0)	13 (54.2)	+
Angina pectoris—no. (%)	7 (50)	0 (0.0)	7 (29.2)	+

+ No *p*-Value determinable due to values of 0.

## Data Availability

Should further information be required regarding the data published herein, interested parties are invited to contact the corresponding authors.

## Supplementary Materials

The following supporting information can be downloaded at: https://www.mdpi.com/article/10.3390/ijms26104562/s1.
