# Supplementary material for: Persistent Monocytic Bioenergetic Impairment and Mitochondrial DNA Damage in PASC Patients with Cardiovascular Complications
Source: Int J Mol Sci. 2025 May 9;26(10):4562. doi: 10.3390/ijms26104562 (PMC12111130; doi:10.3390/ijms26104562)
Supplement: Supplementary file 1 [file ijms-26-04562-s001.zip › ijms-3591529-supplementary.pdf]

Supplementary Material

1 Supplementary Tables

Supplementary Table 1:

RT-qPCR primer sequences used in the study

Gene	Forward primer sequence (5' -3')	Reverse primer sequence (5' -3')
**A-Short**	CCCTAACACCAGCCTAACCA	AAAGTGCATACCGCCAAAAG
**B-Short**	CATGCCCATCGTCCTAGAAT	ACGGGCCCTATTTCAAAGAT
**C-Short**	TCCAACTCATGAGACCCACA	TGAGGCTTGGATTAGCGTTT
**D-Short**	ACTACAACCCTTCGCTGACG	GCGGTGATGTAGAGGGTGAT
**A-Long**	CTGTTCTTTCATGGGGAAGC	AAAGTGCATACCGCCAAAAG
**B-Long**	CATGCCCATCGTCCTAGAAT	TGTTGTCGTGCAGGTAGAGG
**C-Long**	CACACGAGAAAACACCCTCA	CTATGGCTGAGGGGAGTCAG
**D-Long**	CCCTTCGCCCTATTCTTCAT	GCGTAGCTGGGTTTGGTTTA
**tRNA-Leu(UUR)**	CACCCAAGAACAGGGTTTGT	TGGCCATGGGTATGTTGTTA
**Beta-globin**	CACCTTTGCCACACTGAGTGAG	CCACTTTCTGATAGGCAGCCTG
**hPSGL-1**	TGACACCACTCCTCTGACTGGG	CTCCATAGCTGCTGAATCCGTG
**hPECAM-1**	GCAGAGTACCAGGTGTTGGT	GAACAGTTGACCCTCACGAT
**hrplO**	AATCTCCAGGGGCACCATT	CGCTGGCTCCCACTTTGT
